# The influence of simulated exploitation on *Patella vulgata* populations: protandric sex change is size‐dependent

**DOI:** 10.1002/ece3.1872

**Published:** 2016-01-09

**Authors:** Carla D. G. Borges, Stephen J. Hawkins, Tasman P. Crowe, C. Patrick Doncaster

**Affiliations:** ^1^ Centre for Biological SciencesUniversity of SouthamptonLife Sciences Building 85, HighfieldSouthamptonSO17 1BJUK; ^2^ Centro Interdisciplinar de Investigação Marinha e Ambiental, CIIMAR‐PortoLaboratório de Biodiversidade CosteiraRua dos Bragas, 2894050‐123PortoPortugal; ^3^ The Marine Biological Association of the United KingdomThe Laboratory, Citadel HillPlymouth, DevonPL1 2PBUK; ^4^ Ocean and Earth ScienceNational Oceanography CentreUniversity of SouthamptonWaterfront CampusEuropean WaySouthamptonSO14 3ZHUK; ^5^ Earth Institute and School of Biology and Environmental ScienceUniversity College DublinBelfieldDublin 4Ireland

**Keywords:** Human exploitation, limpets, protandry, sex change, size at sex change, size‐selective harvesting

## Abstract

Grazing mollusks are used as a food resource worldwide, and limpets are harvested commercially for both local consumption and export in several countries. This study describes a field experiment to assess the effects of simulated human exploitation of limpets *Patella vulgata* on their population ecology in terms of protandry (age‐related sex change from male to female), growth, recruitment, migration, and density regulation. Limpet populations at two locations in southwest England were artificially exploited by systematic removal of the largest individuals for 18 months in plots assigned to three treatments at each site: no (control), low, and high exploitation. The shell size at sex change (*L*_50_: the size at which there is a 50:50 sex ratio) decreased in response to the exploitation treatments, as did the mean shell size of sexual stages. Size‐dependent sex change was indicated by *L*_50_ occurring at smaller sizes in treatments than controls, suggesting an earlier switch to females. Mean shell size of *P. vulgata* neuters changed little under different levels of exploitation, while males and females both decreased markedly in size with exploitation. No differences were detected in the relative abundances of sexual stages, indicating some compensation for the removal of the bigger individuals via recruitment and sex change as no migratory patterns were detected between treatments. At the end of the experiment, 0–15 mm recruits were more abundant at one of the locations but no differences were detected between treatments. We conclude that sex change in *P. vulgata* can be induced at smaller sizes by reductions in density of the largest individuals reducing interage class competition. Knowledge of sex‐change adaptation in exploited limpet populations should underpin strategies to counteract population decline and improve rocky shore conservation and resource management.

## Introduction

Shorelines have been exploited for food since prehistoric times, as evidenced by shellfish middens at many archaeological sites (Steele and Klein 2008; Álvarez et al. 2011). In many developing countries, subsistence collecting of shellfish has been a principal source of protein (see Hockey et al. 1988; Lasiak 1992). Pressure on resources has increased with the rising global human population, particularly in coastal areas, putting many stocks at risk. Shellfish collection in many countries has changed from a subsistence activity to a highly profitable commercial operation with former subsistence species becoming gourmet items.

Many of the harvested species are mobile grazing herbivores, such as limpets, whose grazing exerts top‐down control of algae in many intertidal ecosystems worldwide (Hawkins and Hartnoll 1983; Hawkins et al. 1992; Moreno 2001; Coleman et al. 2006; Aguilera and Navarrete 2007; Crowe et al. 2011). Limpets are harvested commercially for both local consumption and export in several countries including South Africa (Hockey et al. 1988; Eekhout et al. 1992), the Azores, Madeira and Canaries (Santos et al. 1995; Corte‐Real et al. 1996; Hawkins et al. 2000), Chile (Oliva and Castilla 1986; Duran and Castilla 1989), Mexico, and California (Pombo and Escofet 1996; Fenberg and Roy 2008, 2012).

The effects of humans as top predators on rocky intertidal communities have been the focus of many studies, particularly in South Africa (Hockey et al. 1988; Lasiak 1991, 1992), Chile (Castilla and Duran 1985; Moreno et al. 1986; Oliva and Castilla 1986), Costa Rica (Ortega 1987), and Australia (Catterall and Pointer 1987). Although some studies have analyzed the implications of harvesting in terms of biomass and gonadal outputs (McLachlan and Lombard 1981; Eekhout et al. 1992), most effects of exploitation on populations of intertidal invertebrates have been addressed by comparing sites with and without exploitation, or before and after human exclusion (e.g., Castilla and Duran 1985; Lasiak 1992).

In most cases, collectors prefer larger specimens, putting additional pressure on sequential hermaphrodites – species that change sex, either from male to female (protandric species, such as limpets) or from female to male (protogynic species, such as some labroid fishes). The size‐advantage hypothesis (Ghiselin 1969) has been used for decades to explain the occurrence of sex change. It stated that sex change occurs if an individual reproduces most efficiently (in terms of fertility) as one sex when small or young and most efficiently as the subsequent sex when larger or older, assuming that mortality and growth are the same for both sexes (Ghiselin 1969; Munday et al. 2006). In order to explain variation in timing of sex change, this model was subsequently reframed to include effects of mortality and growth (Warner 1988). Previous experiments had confirmed that the timing of sex change in animals is sensitive to a wide range of factors such as the immediate social environment, the size of an individual relative to others in the social group, sex ratio of social group, and local density (Munday et al. 2006). Some species, however, such as the shrimp *Pandalus borealis* and the limpet *Cymbula oculus*, have a fixed size at sex change despite differences in age structure and mortality rates within and between populations (Munday et al. 2006). Others, such as some protogynic (sequential sex changers from female to male) labroid fishes in the NW Hawaiian Islands have sizes at sex change that vary with species, and within each species, there are differences among reef populations (DeMartini et al. 2005). Other examples exist of variation in timing of sex change between populations, within populations and related to the mating systems (see for details Munday et al. 2006).

Fisheries exploiting hermaphroditic species may disrupt life‐history characteristics, such as operational sex ratios (Alonzo and Mangel 2004, 2005; Heppell et al. 2006; Hamilton et al. 2007). These are often skewed toward the sex that matures first (Allsop and West 2004), and thus the sex that is smaller in size and younger in age (Hamilton et al. 2007). Size‐selective fisheries can therefore be effectively sex‐selective introducing greater bias in already skewed operational sex ratios (Heppell et al. 2006; Hamilton et al. 2007). Such changes for protogynic species can be problematic, leading to sperm limitation and reproductive failure in harvested populations (Alonzo and Mangel 2004; Heppell et al. 2006; Sato and Goshima 2006; Hamilton et al. 2007).

Size and sex‐selective fishing also have predictable consequences for the timing of sex change in sequential hermaphrodites with plastic responses: It is expected that harvest practices will shift the timing of sexual transformation to smaller sizes and younger ages due to the removal of larger sizes predominantly from the second sex (Hamilton et al. 2007). However, compensating this selective harvesting by changing sex at a smaller size will only ensure population persistence if individuals live long enough to attain a size at which sex change can occur (Hamilton et al. 2007). Examples of studies indicating fishing pressure as a cause for reduction in the size at sex change are available for shrimp and fish (Hannah and Jones 1991; Platten et al. 2002; Hawkins and Roberts 2003; Hamilton et al. 2007).

Some management models incorporate closed areas (spawning or other marine protected areas) and quotas (on specific size classes) to maintain sex ratios, preserve age structure, prevent sperm limitation, enhance yield, and restrict evolutionary changes in response to fishing, such as shifts to earlier maturation (Buxton 1993; Alonzo and Mangel 2004; Baskett et al. 2005; Heppell et al. 2006; Hamilton et al. 2007). Others emphasize the capacity for sex‐change rules (endogenous vs. exogenous cues) to influence stock dynamics, spawning‐per‐recruit measures, and fertilization rates (see Alonzo and Mangel 2005; Hamilton et al. 2007). Therefore, in addition to minimum size limits used to prevent recruitment, overfishing management policies should also consider slot limits (i.e., minimum and maximum size limits) for sex‐changing fisheries (Hamilton et al. 2007). For protogynic fish, slot limits will help to prevent sperm limitation by reducing the removal of large males, but will also help ensure that large females, with exponentially greater fecundity, will contribute to future generations (Hamilton et al. 2007). The rules governing sex change should be clarified in further studies and considered in management policies for hermaphroditic species (Hamilton et al. 2007) that should not neglect the effect of top predators on sex‐changing prey (DeMartini et al. 2005).

Density and sex ratio have been shown to influence sex change within the order Patellogastropoda (see Wright and Lindberg 1982; Lindberg and Wright 1985; Wright 1989; Collin 2013). In general, protandric limpets change sex at smaller sizes under depletion of females and lower densities. The reported period of time to change sex can vary from 5 months in *Crepidula norrisiarum* (Warner et al. 1996) to 1 year in *Lottia gigantea* (Wright 1989). Sex change in protogynic fish can occur within a 6‐month period (Hawkins and Roberts 2003), while in a protandric simultaneous hermaphroditic shrimp, time to change sex was 9.8 weeks in group treatments and 9.4 weeks in individual treatments (Baldwin and Bauer 2003).

**Figure 1:**
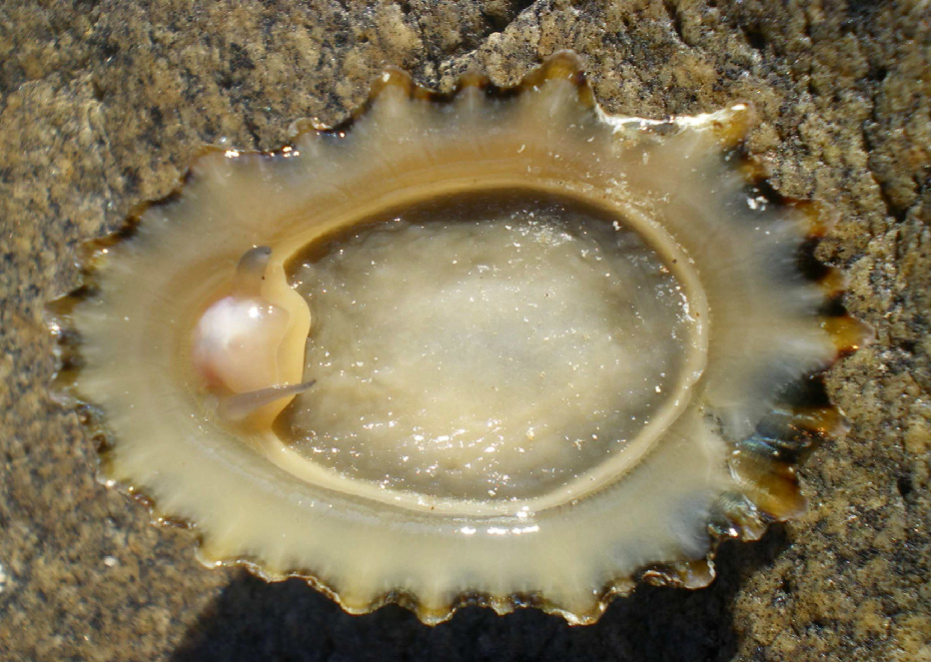
Ventral view of *Patella vulgata*.

Our paper describes a novel field experiment designed to simulate human exploitation of limpets (*Patella* spp.) in order to evaluate its plasticity in the population ecology of *Patella vulgata* (a model target species, Fig. 1). The natural abundance of *P. vulgata* and occurrence of sex change provides a model for investigating the consequences of predation by humans upon its demography and patterns of sex change. As several other limpets known to be protandric (e.g., *P. ulyssiponensis, P. aspera, P. caerulea, P. ferruginea*,* C. oculus*) are at risk from overexploitation and habitat degradation, understanding sex change in the Patellidae can inform future management and conservation strategies for rocky shores in Europe and worldwide. Although it changes sex from male to female as it grows in size, in common with other exploited species (e.g., some *Patella* spp., *C. oculus,* and *L. gigantea*), few studies have addressed experimentally the potential implications of human exploitation on demographic sex ratios (but see Rivera‐Ingraham et al. 2011; Fenberg and Roy 2012; for descriptive data on protected and exploited populations). This reflects the difficulty of following sex change in an individual through its life (but see Wright and Lindberg 1982; Le Quesne and Hawkins 2006). Unlike the genus *Crepidula*, where sexes can be recognized by external visual inspection, *P. vulgata* has no visible external sexual characters, and removing an individual from the substratum often greatly impacts on its probability of survival. Determining and following sex over time in individual limpets therefore usually involves high mortality rates (Le Quesne and Hawkins 2006).

Our experimental design is innovative in that it allows comparisons of two rates of limpet exploitation of originally unexploited populations and this is the first experiment to simulate limpet size‐selective exploitation continuously at a comparatively large scale. Prior investigations have confirmed that some *Patella* spp. are able to undergo sex change between consecutive spawning seasons (Le Quesne and Hawkins 2006; Guallart et al. 2013). Sex reversal (sex change back from female to male) has also been reported for *P. vulgata,* where one of eight females reverted to males during a 12‐month period (Le Quesne and Hawkins 2006), while for *P. ferruginea* two of 21 females were documented to be male on the subsequent spawning season (Guallart et al. 2013). Limpet populations were exploited by systematic removal of larger specimens over an 18‐month period, which included two consecutive spawning seasons, during which records were kept of limpet sex‐change responses, growth size, food availability, recruitment, migrations, and densities.

The hypothesis tested was that selective removal through time of the largest size classes of limpets would drive a change at phenotypic level (i.e., a plastic response), in the timing and magnitude of sex change. Thus, removal of larger limpets (predominantly females) could possibly induce an earlier (in size) sex change in the resident males. *Patella vulgata* is a late autumn–winter breeder (Orton et al. 1956) that passes through a neuter immature stage which can be followed by a male and subsequently a female stage, after resting as a neuter between breeding seasons from January to June. Fertilization occurs in the water column where both female and male gametes are expelled during spawning. Some individuals may remain the same sex throughout their lives, as both small females and large males are occasionally found. As in some fish species, it could be the case that *P. vulgata* females could also arise from immature neuters that bypassed the male phase (Guallart et al. 2013; see Fennessy and Sadovy 2002; de Girolamo et al. 1999; Allsop 2003 for the early maturers concept). With the removal of the larger individuals mainly depleting the female population, it was hypothesized that the mean size of remaining males would be smaller at the end of the experiment due the transition of the larger males to females. Consequently, the mean sizes of immature neuters and females would also be smaller in treatments than in the controls. Sex change was predicted to occur at a smaller size resulting in smaller mean sizes of the different sexual stages in treatment relatively to control plots. It was further hypothesized that the sex change would vary under different levels of exploitation, being size‐dependent and occurring at smaller body sizes where the simulated exploitation was higher (and hence fewer female limpets were left). Any response in body size at the time of sex change could also alter the relative abundances of the sexual stages. However, the loss of females under exploitation treatments would be compensated by a reduction in numbers of neuters and males due to their direct passage from neuter to female (bypassing the male stage) or indirect passage from neuter to male to female. These would be predicted to switch to the next sexual stage faster (and hence at smaller sizes) relative to the control plots. Reduction in numbers of males could in turn be compensated by neuters changing early to males with densities being offset by recruitment. In order to explain the possible patterns arising from the experiment, we tracked limpet growth and potential migration between treatment plots.

## Materials and Methods

### Study organisms and locations

In the study area, two other species co‐occur with *P. vulgata* in the mean tide level but at lower abundances: *Patella depressa* (<8%) and *P. ulyssiponensis* (<2%). These were included in the removals as their unequivocal identification requires observation of the ventral side. Given the low abundances of the other species during the experiment, we present only responses of *P. vulgata* populations. The limpet *P. vulgata* is the dominant grazer in the mid‐intertidal zone of the southwest of England (Jenkins et al. 2005; Coleman et al. 2006) and is distributed across the wave exposure gradient. *Patella depressa* also occurs on open rock, and *P. ulyssiponensis* can be encountered in mid‐shore depressions and rock pools but is far more abundant lower on the shore. These species feed primarily on the microbial films that coat rocky shores (Hawkins et al. 1989), although recent work has shown that they can also consume adult macroalgae (Davies et al. 2007).

The phenomenon of protandry in the Patellidae has been known since the early work of Orton (1919), and colleagues (Dodd 1956; Orton et al. 1956) and cytological work by Bacci (1975). *Patella vulgata* is the sex‐changing species that has been most widely studied in Europe, although data exist also for sex change in the edible *P. ulyssiponensis* (Thompson 1979), *P. caerulea* (Montalenti 1958), *P. ferruginea* (Espinosa et al. 2009a, 2009b; Guallart et al. 2013), and *Patella rustica* (Bacci 1975). In most of the cases, knowledge of sex‐change patterns comes mainly from the interpretation of sex with size data from field collections, although sex change has been tracked in individual *P. vulgata* (Le Quesne and Hawkins 2006) and *P. ferruginea* (Guallart et al. 2013).

The study was carried out at Constantine (50°31′52.02″N, 5°01′33.32″W) and Trevone (50°32′40.48″N, 4°58′50.08″W) on the coast of north Cornwall, United Kingdom (UK), identified as being little disturbed by people, having similar tidal ranges and shore extents and where limpets are not human target species as in most of the UK today. These locations both have a slate bed rock, although Constantine is more exposed to wave and wind action than Trevone. Despite the exposure of this location, its shallow profile causes a reduction in wave energy up the reef. The study locations had considerable differences in assemblage structure: Constantine can be considered an exposed shore with a *Mytilus* spp.‐dominated shore community, while the mid‐shore at Trevone can be considered a moderately exposed shore with barnacles and a patchy *Fucus*‐dominated community.

### Experimental design and procedures

The experiment was established on each site as a block design, with three levels of experimental exploitation of limpets: Control, Low, and High. At each of the two locations, three adjacent blocks of shore each about 20 m wide were defined at the mean tide level. In each block, three unfenced treatment plots of 3 × 3 m (one of each treatment) were randomly placed on the available rock surface. Plots were sited away from large rock pools where possible to minimize habitat heterogeneity and the presence of *P. ulyssiponensis*. As limpets often forage up to 1.2 m from their home scar, to ensure independence of plots, they were all separated by at least 5 m and no limpet was observed to move from one plot to another. The experiment ran for 18 months from March 1999 to October 2000 and included five limpet removal events, one every 3 months. No limpets were removed from the controls (C). At the low exploitation treatments (L), all limpets with a base length of shell ≥25 mm were removed from half of the available area, by sampling from alternate 18 squares in a grid of 0.5 × 0.5 m quadrats covering the plot, starting from one randomly attributed quadrat. In plots assigned to the high exploitation treatment (H), all limpets with a base length shell ≥25 mm were removed at each visit. The simulated exploitation treatments involved removing the largest limpets at two different rates in accordance with what happens in most exploited limpet populations, where larger sizes are preferentially removed by collectors (e.g., Hockey et al. 1988; Espinosa et al. 2009a, 2009b; Fenberg and Roy 2012). After measuring each limpet with callipers, it was knocked from the rock with a screwdriver and hammer. All removed limpets were stored frozen and processed in the laboratory where they were measured again and sexed if not in the resting stage following Orton et al. (1956) as described below. In conjunction with each visit to remove limpets, data were collected to enable estimates of:

1. Limpet sex‐change responses, by analyzing at the end of the experiment: mean sizes of sexual stages; size at sex change following Collin (2006); relative abundances of sexual stages; cumulative size frequencies; and relative size at sex change.
2. Limpet growth through increments in maximum shell length.
3. Food availability through the assessment of microalgal film abundance.
4. Recruitment and limpet migrations into and out of the experimental plots.

The collection of data and derivation of each of these response variables are described below.

### Limpet sex‐change responses

At the end of the experiment, at the completion of the breeding season, all limpets, regardless of their size, were removed from the plots under the different exploitation pressures. The limpets were frozen and their sex determined in the laboratory following Orton et al. (1956) by direct observation of the gonad aided with a microscope when visual inspection was inconclusive. This required removal of the foot to expose the gonad which is generally pink or orange in males and green or brown in females. Immature neuters lack a fully developed gonad.

The influence of the exploitation treatment on sex change was tested with the following response variables: mean limpet shell sizes, size at sex change following Collin (2006), relative abundances of sexual stages, and cumulative sex–size frequencies. Cumulative size frequencies for the different sexual stages in the different treatments were plotted to illustrate the shifts in size and numbers of the sexual stages.

The response variable of size at sex change was represented by *L*_50_, the size in millimeters at which 50% of non‐neuter individuals were female (Collin 2006). Neuters were excluded because they present no sexual characteristic. *L*_50_ was estimated for *P. vulgata* using the logistic regression approach adopted by Allsop and West (2003). For each treatment plot, the value of *L*_50_ was extrapolated using Minitab 16, State College, PA, USA from the following logistic regression on the odds ratio of males to females as a function of body size:

Log[P(male)/P(female)]=a−b×Size

where *a* and *b* are the intercept and slope of the linear regression. The *L*_50_ is the body size *a*/*b* at which an individual is as likely to be male as female, and therefore, the log‐ratio equals zero.

### Responses of limpet growth and micro‐algae film abundance

Between 30 and 40, *P. vulgata* individuals with shell lengths of 17.5 ± 2.5 mm (between 15 and 20 mm) were double tagged with numbers, to allow individual recognition, at each treatment plot at the beginning of the experiment at Constantine and Trevone. Double tags were used to minimize the probability of tag losses that could be due to predation, mortality of individuals, or poor fixation of the tags. Growth in shell size was measured from 1999 to 2000 in spring, summer, and autumn. Due to losses of individuals and tags, the number of tagged individuals used to calculate the growth rate of each plot was reduced to the minimum number of six individuals found in all three sampling seasons (spring, summer, autumn) in 1999 (considered representative of the whole experiment, as the tagged individuals found in 2000 were insufficient). Growth rates were calculated for each individual from its total change in length between spring and autumn 1999, expressed in millimeters per month.

The abundance of microalgal film was compared between different rates of exploitation in June 2000, using chlorophyll *a* as an index of standing crop for the plots from the two shores. The surface area of each rock sample was determined by image analysis, and the level of chlorophyll *a* per unit area of rock surface was calculated using the following equation (Thompson et al. 1999):

$$\text{Chlorophyll}\;a\;\text{concentration}\;\mu g\cdot {\text{mm}}^{-2}=\frac{13.0\times {Å}_{\mathrm{net}}\times \nu }{d\times a}$$

where Å_net_ = net absorbance of chlorophyll solution (Å665–Å750), *ν *= volume of solution (mL), *d* = path length of cell (mm), *a* = surface area of sample (mm^2^).

Initially, a random sample was taken from each plot of 18 rock chips with a minimum surface area of 200 mm^2^ and free of barnacles and encrusting algae, using a hammer and a fine chisel (blade size, 10–20 mm). As no differences in standing crop were detected, another sample of nine rock chips was randomly taken from within the edge (500 mm) of each plot and another nine from the center of each plot to investigate differences between the edges and center of the plots.

### Limpet recruitment and migratory responses

Movement patterns were tested in the three sampling seasons of spring, summer, and autumn in 1999, with paint loss in subsequent months precluding further tests. Before each removal procedure, all resident limpets were painted with enamel paint, and then, incoming limpets were painted with a different color to establish which limpets were marked in different seasons (spring: white, summer: yellow, autumn: green) in order to look for patterns of movements into and out of experimental plots. A pilot study indicated that the paint did not cause any mortality to the limpets due to extra predation or toxicity. Based on a pilot study, 10 random quadrats, each of 0.5 × 0.5 m, were used in each treatment plot at each sampling time to assess densities, sizes, and the color of paint on shells, of the different limpet species. Recruits into treatment plots (designated *Patella* spp. since could not be separated by species) were registered for summer and autumn 1999, and no limpet was observed to have moved out of its original plot.

### Statistical analysis

A two‐factor split‐plot design was used to test the hypothesis that selective removal through time of large limpets would influence timing and magnitude of several biological processes related to the sex change, measured at the end of the experiment (model 1). The design had three levels of the fixed Treatment factor (T_3,_ number of levels indicated in the subscript) in each of three random Blocks ($B_{3}^{\prime }$) nested in each of two levels of a random Location factor ($L_{2}^{\prime }$). This design was analyzed with the ANOVA model:

(model 1) $$Y=T|B^{\prime }\left(L^{\prime }\right)$$

where henceforth a prime represents a random factor, vertical line means “crossed with” and parenthesis means “nested in”.

The use of Location as a random factor was dictated by the aim to extrapolate the final results to the southwest coast of England from which the two locations were selected. Although the treatment effect was tested with only 2 error degrees of freedom, if the interaction between Location and Treatment was not detectable at *α *= 0.25, it was pooled with the error term to provide a more powerful test for the treatment effect with 10 error degrees of freedom (post hoc pooling, following Underwood 1997).

In order to test the prediction that intensification of the rate of exploitation would reduce size at sex change, therefore reducing abundance and mean sizes of different sexual stages, model 1 was expanded to include a fixed cross‐factor of sexual stage (S_3_) with levels of neuter, male, female. Stage‐specific differences in body size and abundance in response to selective predation were investigated with the three‐factor split‐plot ANOVA model:

(model 2) $$Y=S|T|B^{\prime }\left(L^{\prime }\right)$$

Two sets of planned a priori contrasts were used to detect differences between Treatment and Stage effects. Treatment contrasts partitioned the sums of squares of the exploitation treatment, with a first contrast of no exploitation (NE: control) and exploitation (EX: low and high pooled), and a second orthogonal contrast of exploitation levels (low vs. high). Stage contrasts partitioned the sums of squares of sexual stages, with a first contrast of no sex (NS: neuters) to sexually mature individuals (SE: males and females pooled) and a second orthogonal contrast between the sexes (males vs. females).

As the removal of limpets increased the available space, model 1 was applicable to the investigation of differences in the microalgal film and consequently its impact on limpet growth. The model was augmented with a fixed cross‐factor of Distance (*D*_2_). This factor tested for differences in microalgal film abundance from the edge to the center of the plot, which could be due to outsider limpets foraging on the edge of the plots. The design was a three‐factor split‐plot ANOVA model:

(model 3) $$Y=D|T|B^{\prime }\left(L^{\prime }\right)$$

All three‐factor ANOVAs (models 2 and 3) run in Minitab 16 used a restricted “model 2” analysis of random factors, which assumed that all mean squares for Block‐by‐Treatment interactions measured the same quantity, being pooled into a single variance component for the error term (Doncaster and Davey 2007).

Treatment effects on the relative frequencies of males and females were tested with a generalized linear mixed model (GLMM) using a binomial error structure, with Site (two levels) and Block (three levels) as random factors, Size as a covariate, and Treatment (three levels) as a fixed factor. As Block and Location accounted for negligible variation, the model was simplified to a generalized linear model (GLM) using a binomial error distribution with covariate of Size and fixed factor of Treatment. This analysis was carried out with R version 3.2.0 (R Core Team 2015). The fixed factors were tested using the function “Anova” from “car” package, after which we rescaled the logistic coefficients to probability interval [0, 1] using the function “plogis”.

Whether the relative size at sex change is invariant across the limpet populations at the two shores was tested by plotting ln(*L*_50_) against ln(*L*_max_) for each treatment plot from both shores on the null prediction of a slope of 1.0. *L*_max_ was calculated as the top 2.5% quantile for each size frequency data. The averaged value across treatments of *L*_50_/*L*_max_ will be the relative averaged size of sex change.

For each location, nine pairwise Kolmogorov–Smirnov (K‐S) tests with Bonferroni correction of *α* (Sokal and Rohlf 1998) were performed on cumulative size frequency distributions of neuters, males and females for each of the three levels of treatment.

## Results

### Limpet removals during the experiment

**Figure 2:**
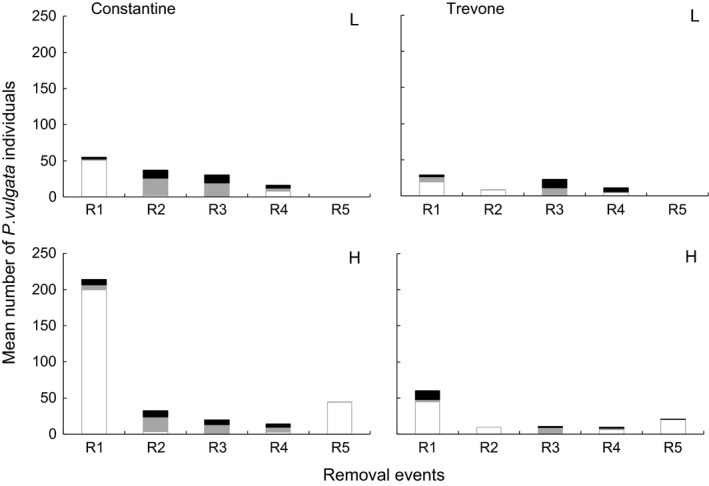
Mean numbers per 3 × 3 m plot of *Patella vulgata* neuters (clear bars), males (gray bars), and females (black bars) removed since the beginning of the experiment (R1) until the last removal (R5) before the end of the experiment at Constantine (left graphs) and Trevone (right graphs) in low (L) and high (H) treatments.

During the experiment, the relative abundances of sexual stages of the removed limpets varied accordingly to the phase of the breeding season (Fig. 2): At the beginning of the experiment in March 1999, during the resting phase most *P. vulgata* were in the neuter stage and these comprehended most of the removed limpets particularly at Constantine. In the following removal events, the numbers of removed limpets were much reduced.

### Sizes of limpet sexual stages at the end of the experiment

**Figure 3:**
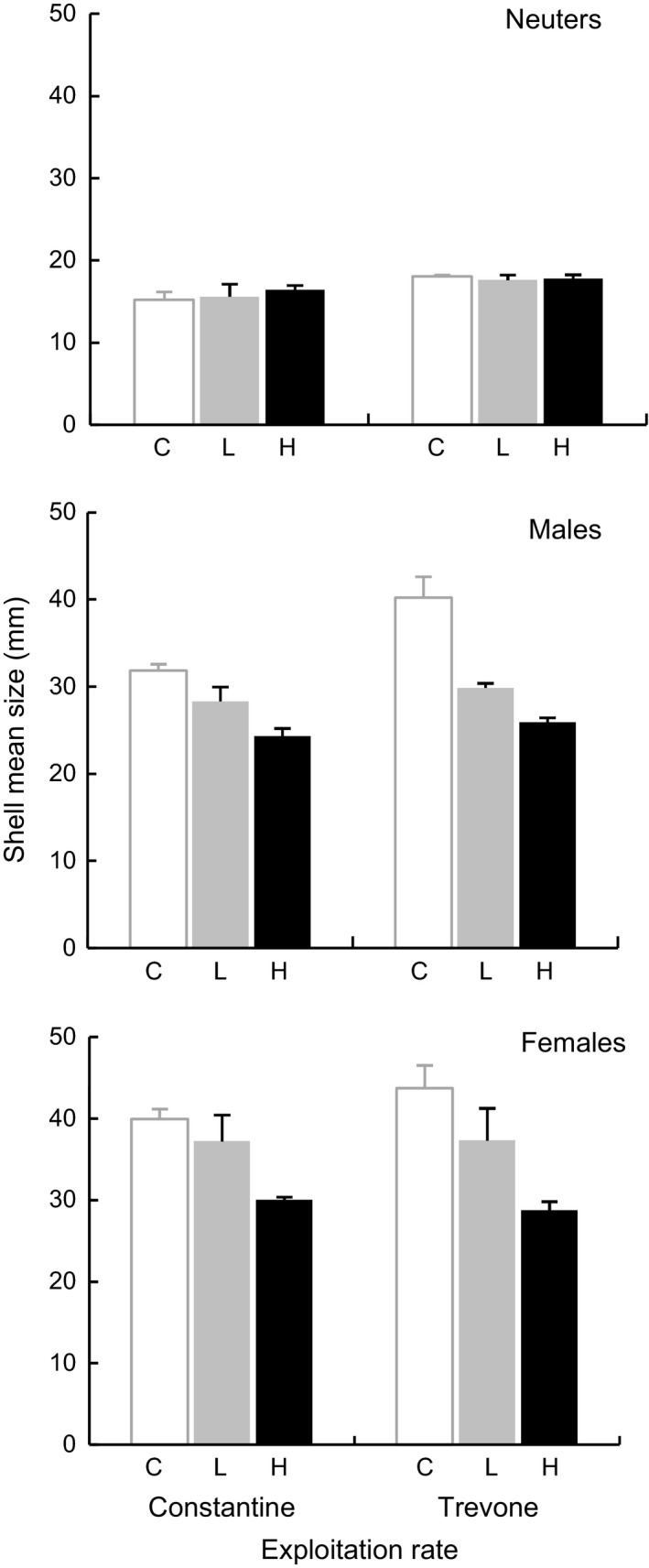
Mean body sizes (±SE, *n *=* *3 plots per sample) of *Patella vulgata* neuters (top row), males (middle row) and females (bottom row) at the end of the experiment, by treatment (control: C, low exploitation: L, high exploitation: H) at Constantine (*Mytilus*‐dominated) and Trevone (barnacle/*Fucus*‐dominated).

The mean shell size at the end of the experiment in the unexploited control differed from the two exploitation treatments, which themselves differed between each other, both as main effects and in the interaction with sexual stage (Fig. 3, Table 1). There were no detectable differences by location. *Patella vulgata* was larger in the absence of exploitation (control: 31.5 ± 5.0 mm) than with exploitation (Table 1: low and high exploitation levels pooled: 25.8 ± 2.2 mm, *F*_1,2_ = 40.46, *P *=* *0.024). Among treatments, limpets were significantly smaller under high exploitation (23.9 ± 2.3 mm) compared to low exploitation (Table 1: 27. 7 ± 3.8 mm, *F*_1,2_ = 19.82, *P *=* *0.047). Thus, the experimental manipulation was effective in removing large limpets.

**Table 1:** Response of *Patella vulgata* mean size at the end of the experiment (ANOVA model 2: *L*′ = location, *B*′(*L*′) = block nested in location, *T* = treatment, *S* = stage) at different sexual stages (neuters; males; females) by exploitation treatment (control; low; high) and location. Exploitation orthogonal contrasts are shown partitioning the treatment SS between no exploitation (NE: control) and exploitation (EX: pooled low + high) and between exploitation levels (low vs. high). Stage orthogonal contrasts are shown partitioning the stage SS between not sexed (NS: neuters) and sexually mature individuals (SE: males and females pooled) and between the sexes (males vs. females). Terms with *P *<* *0.05 are in bold

Source	df	Seq SS	Seq MS	*F*	*P*
Between subjects					
*L*′	1	32.42	32.42	1.45	0.295
*B*′(*L*′)	4	89.39	22.35	–	
Within subjects					
*T*	2	388.78	194.39	30.16	**0.032**
NE vs. EX	1	260.94	260.94	40.46	**0.024**
Low vs. high	1	127.84	127.84	19.82	**0.047**
*L*′ × *T*	2	12.89	6.45	1.08	0.353
*L*′ × (NE vs. EX)	1	11.80	11.80	1.97	0.170
*L*′ × (low vs. high)	1	1.09	1.09	0.18	0.674
*S*	2	3379.62	1689.81	286.63	**0.003**
NS vs. SE	1	2997.63	2997.63	508.07	**0.002**
Males vs. females	1	381.99	381.99	64.74	**0.015**
*S* × *L*′	2	11.79	5.90	0.98	0.385
(NS vs. SE) × *L*′	1	1.78	1.78	0.30	0.588
(males vs. females) × *L*′	1	10.01	10.01	1.67	0.206
*S* × T	4	251.79	62.95	123.43	**<0.001**
*S* × (NE vs. EX)	2	146.71	73.36	143.84	**<0.001**
*S* × (low vs. high)	2	105.08	52.54	103.02	**<0.001**
(NS vs. SE) × *T*	2	227.87	113.94	223.41	**<0.001**
(males vs. females) × *T*	2	23.92	11.96	23.45	**0.006**
*S* × *L*′ × *T*	4	2.03	0.51	0.08	0.986
*S* × *L*′ × (NE vs. EX)	2	1.29	0.65	0.11	0.896
*S* × *L*′ × (low vs. high)	2	0.74	0.37	0.06	0.942
(NS vs. SE) × *L*′ × *T*	2	1.28	0.64	0.11	0.899
(males vs. females) × *L*′ × *T*	2	0.75	0.37	0.06	0.940
Residual error	32	191.58	5.99		

The treatment effect on shell size depended on sexual stage (Table 1: *F*_4,4_ = 123.43, *P *<* *0.001). Mean shell size of *P. vulgata* neuters changed little under different levels of exploitation, while males and females both decreased markedly in size with exploitation (Fig. 3). The differential influence of treatments on the mean sizes of sexual stages was most evident between immature neuter and mature stages (Fig. 3, Table 1: (NS vs. SE) × T, *F*_2,4_ = 223.41, *P *<* *0.001), but was also detected between the sexes (Table 1: (males vs. females) × T, *F*_2,4_ = 23.45, *P = *0.006).

Overall, neuters (16.8 ± 0.5 mm) were smaller than mature individuals (Fig. 3, Table 1: male and females pooled: 33.1 ± 1.8 mm, *F*_1,2_ = 508.07, *P *=* *0.002), and males (30.1 ± 2.3 mm) were significantly smaller than females (36.2 ± 2.4 mm) (Fig. 3, Table 1: *F*_1,2_ = 64.74, *P *=* *0.015).

### Size at sex change (*L*_50_)

**Figure 4:**
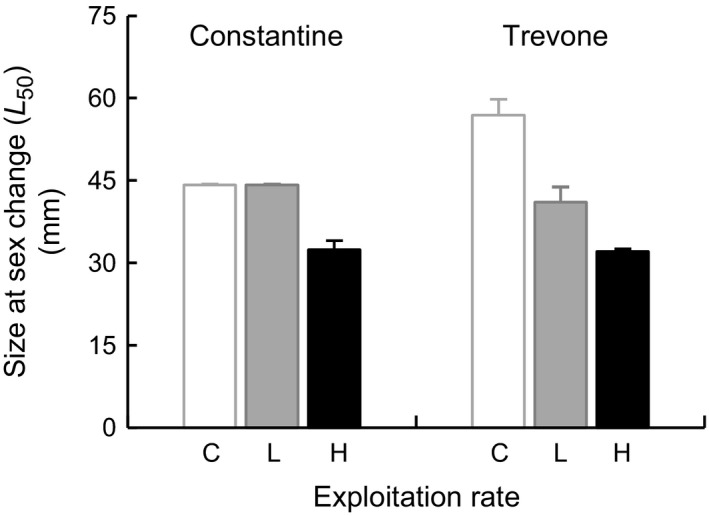
*Patella vulgata* size at sex change (*L*_50_) (±SE,* n *=* *3 plots per sample) at the end of the experiment by treatment plot (codes and locations as for Fig. 2 legend).

The average *L*_50_ extracted from the logistic regression on each treatment plot shows that the size at sex change decreased with increased exploitation (Fig. 4: control: 50.5 mm ± 6.4, low: 42.6 mm ± 1.5, high: 32.2 mm ± 0.2). This result was corroborated by the GLM showing significant effects of Size and Treatment on Sex (relative frequencies of males and females) but no influence of Location or Block. (Table 2a,b). In effect, increased Size increased the probability of being female; but the effect of Treatment was more important, with the probability of being female increasing in the High exploitation treatment relative to the Low exploitation treatment and the Low exploitation treatment relative to the Control (Table 2c).

**Table 2:** (a) GLM output of influences on the relative frequencies of males and females due to shell size (covariate) and treatment (three level factor: C, L, H). Block and Location accounted for negligible variation in the initial GLMM model, so it was simplified to a generalized linear model (GLM). Factor codes as for Table 1, and Si = size. (b) Output of the “Anova” function from the “car” package on the GLM model. (c) Rescaled logistic coefficients of the GLM to probability interval [0,1]. Total number of observations = 2016. Terms with *P *<* *0.05 are in bold

(a)			
	Estimate	Standard error	Pr(>|*z*|)
C (Intercept)	−4.09	0.27	**<0.001**
*L*	0.66	0.13	**<0.001**
*H*	1.17	0.15	**<0.001**
Si	0.08	0.01	**<0.001**

(b)			
	*χ* ^2^	df	Pr(>*χ* ^2^)
T	63.06	2	**<0.001**
Si	212.75	1	**<0.001**

(c)			
C (Intercept)	*L*	*H*	Si
0.02	0.66	0.76	0.52

### Abundances of limpet sexual stages at the end of the experiment

**Figure 5:**
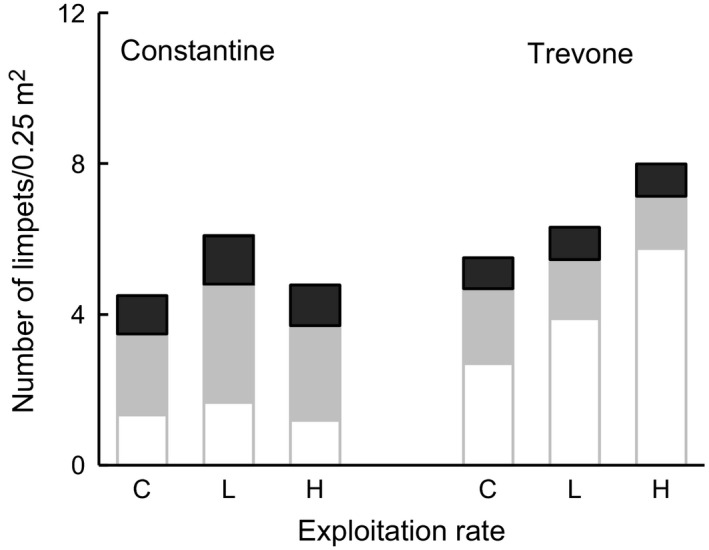
Total number (scaled to sampled quadrat) of *Patella vulgata* neuters (clear bars), males (gray bars) and females (black bars) removed at the end of the experiment, by treatment (codes and locations as for Fig. 2 legend).

At the end of the experiment, the relative abundance of sexual stages differed between locations. Neuters were more abundant at Trevone than at Constantine and their abundance increased with increased exploitation (Fig. 5). Statistical analysis showed an interaction of the NS vs. SE contrast with location and treatment (Table 3: *F*_2,32_ = 4.40, *P *=* *0.021) interaction.

**Table 3:** Response of *Patella vulgata* numbers per quadrat at the end of the experiment (ANOVA model 2: Factor codes as for Table 1) at different sexual stages (neuters; males; females) by exploitation treatment (control; low; high) and location. Stage orthogonal contrasts as for Table 1. Terms with *P *<* *0.05 are in bold

Source	df	Seq SS	Seq MS	*F*	*P*
Between subjects					
*L*′	1	3.28	3.28	0.87	0.403
B′(*L*′)	4	15.02	3.75	–	
Within subjects					
*T*	2	2.27	1.13	0.94	0.515
*L*′ × T	2	2.41	1.20	1.54	0.230
*S*	2	28.77	14.39	0.84	0.544
NS vs. SE	1	17.16	17.16	1.00	0.423
Males vs. females	1	11.61	11.61	0.68	0.496
*S* × *L*′	2	34.27	17.14	21.97	**<0.001**
(NS vs. SE) × *L*′	1	33.27	33.27	42.65	**<0.001**
(males vs. females) × *L*′	1	1.00	1.00	1.28	0.266
*S* × T	4	4.69	1.17	0.63	0.668
(NS vs. SE) × T	2	4.54	2.27	1.22	0.386
(males vs. females) × T	2	0.15	0.75	0.40	0.695
*S* × *L*′ × T	4	7.45	1.86	2.38	0.072
(NS vs. SE) × *L*′ × T	2	6.86	3.43	4.40	**0.021**
(males vs. females) × *L*′ × T	2	0.59	0.29	0.37	0.693
Residual error	32	25.01	0.78		

### Cumulative size frequencies at the end of the experiment

**Figure 6:**
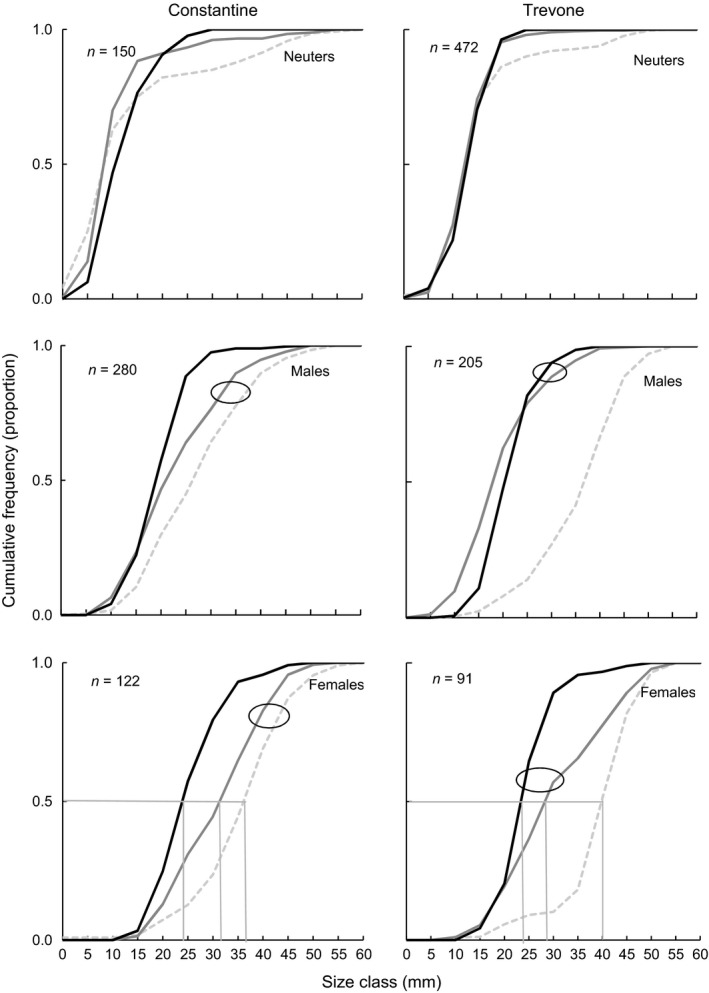
Cumulative frequencies per 3 × 3 m treatment plot for *Patella vulgata* neuters (top row), males (middle row), and females (bottom row) at the end of the experiment by no‐exploitation control (gray broken line), low exploitation (gray line), and high exploitation (black line), at Constantine (left) and Trevone (right). *n *= number of individuals per plot. The observed *L*_50_ size class is the size class with 0.5 males and females. Circles group similar size distributions.

Both locations showed evidence of the reduction in number of larger individuals from low exploitation treatments to high exploitation treatments, and relative to the control (Fig. 6). At Constantine, both male and female size frequency distributions from High exploitation plots differed from those in Control and Low exploitation plots (K‐S test, *α* < 0.02), which were similar to each other (K‐S test, *α* > 0.02). At Trevone, both male and female distributions from Control plots differed from those in Low and High exploitation plots (K‐S test, *α* < 0.02), which were similar to each other (K‐S test, *α *> 0.02). This suggests that while at Constantine only a high level of removal of limpets had an impact on the distributions of both males and females compared to those in the Control treatment plots, at Trevone a low‐intensity removal of limpets sufficed to change the distributions of males and females compared to those the Control plots. No differences were detected (K‐S test, *α *> 0.02), however, in the distribution of neuters between the three treatments at either of the locations.

The cumulative frequency curves also show a decrease with exploitation of the size class with 50% females (Fig. 6).

### Relative size at sex change (*L*_50_ */L*_max_)

**Figure 7:**
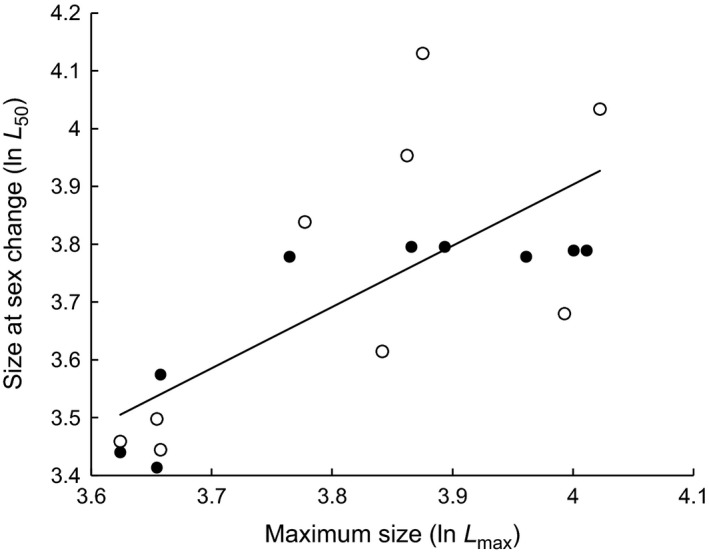
Logarithmic plot of size at sex change (*L*_50_) versus maximum size (*L*_max_) for *Patella vulgata* of the treatment plots from Constantine (●) and Trevone (○) ln(*L*_50_) = 1.06 × ln(*L*_max_) – 0.334.

The relation between *L*_50_ and *L*_max_ shows that increases in *L*_max_ result in increases in the *L*_50_ for each location separately (Constantine: *r* ^2^ = 0.73, slope = 0.893; Trevone: *r* ^2^ = 0.51, slope = 1.274) with a slope not significantly different from unity for the two locations combined (Fig. 7: *r* ^2^ = 0.50, slope = 1.059 ± 0.526 confidence intervals at *P *=* *0.05). This suggests that the ratio *L*_50_ */L*_max_ is invariant across the two populations of *P. vulgata* and that limpets were changing sex at a constant proportion of their maximum size. The average value across the plots of *L*_50_ */L*_max_ was of 0.90 indicating that *P. vulgata* changed sex when reaching 90% of its maximum size.

### Limpet growth and micro‐algae film abundance

**Figure 8:**
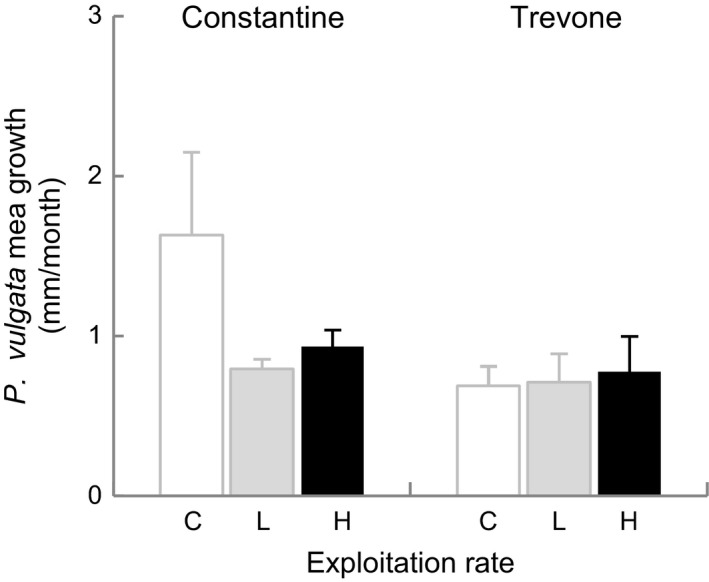
Estimated *Patella vulgata* mean growth per month (±SE, *n *= 3 plots per sample) by treatment (codes and locations as for Fig. 2 legend) from spring to autumn 1999.

**Figure 9:**
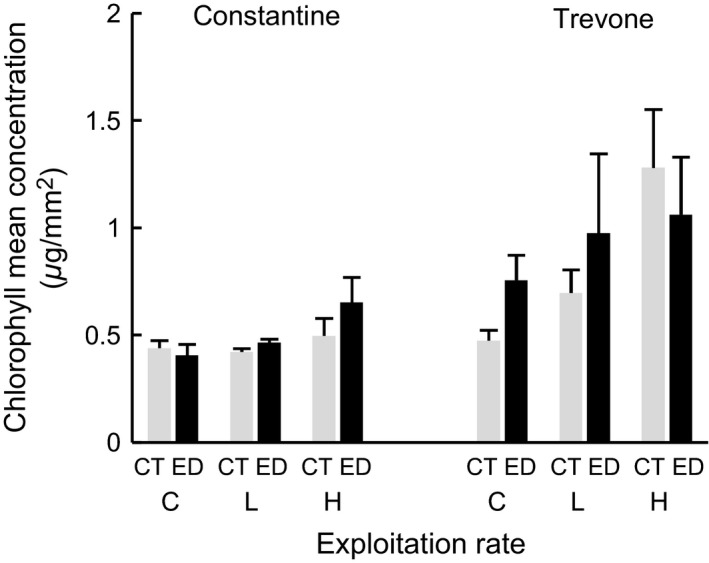
Chlorophyll mean abundance (±SE, *n *=* *3 plots per sample) at the center (CT: gray bars) and edge (ED: black bars) of plots under treatments in June 2000. Codes and locations as for Fig. 2 legend.

No differences (*P *>* *0.05) were detected in estimated growth of *P. vulgata* from spring to autumn 1999, either under different levels of exploitation or between locations (Fig. 8). The available microalgal food sampled as chlorophyll abundance in June 2000 did not differ between plots under different levels of exploitation or between locations (*P *>* *0.05). There was, however, a tendency for increase in microalgal film with increase in exploitation, particularly at Trevone (Fig. 9). Four months before the end of the experiment, microalgal food responded to exploitation differently at the two locations (Fig. 9, Table 4: L × T: *F*_2,20_ = 4.00, *P *=* *0.035). Center and edge plots exhibited differences in chlorophyll abundance by Location and Treatment: Plot edges under high exploitation at Trevone had lower chlorophyll abundance than corresponding plot centers (Fig. 9, Table 4: Di × L′ × (low vs. high): *F*_1,20_ = 4.67, *P *=* *0.043).

**Table 4:** ANOVA on chlorophyll abundance in June 2000, by distance from plot center, treatment, and location (model 5: Factor codes as for Table 1, and Di = distance). Orthogonal contrasts as for Table 1. Terms with *P *<* *0.05 are in bold

Source	df	Seq SS	Seq MS	*F*	*P*
Between subjects					
*L*′	1	1.39	1.39	3.86	0.121
B′(*L*′)	4	1.44	0.36	–	
Within subjects					
*T*	2	0.77	0.38	3.17	0.240
NE vs. EX	1	0.45	0.45	3.75	0.192
Low vs. high	1	0.32	0.32	2.67	0.244
*L*′ × *T*	2	0.24	0.12	4.00	**0.035**
*L*′ × (NE vs. EX)	1	0.18	0.18	6.00	**0.024**
*L*′ × (low vs. high)	1	0.06	0.06	2.00	0.173
Di	1	0.06	0.06	6.00	0.247
Di × *L*′	1	0.01	0.01	0.33	0.572
Di × *T*	2	0.06	0.03	0.30	0.771
Di × (NE vs. EX)	1	0.01	0.01	0.10	0.782
Di × (low vs. high)	1	0.05	0.05	0.50	0.553
Di × *L*′ × *T*	2	0.21	0.10	3.33	0.056
Di × *L*′ × (NE vs. EX)	1	0.07	0.07	2.33	0.143
Di × *L*′ × (low vs. high)	1	0.14	0.14	4.67	**0.043**
Residual error	20	0.58	0.03		

### Limpet recruitment and migratory responses

**Figure 10:**
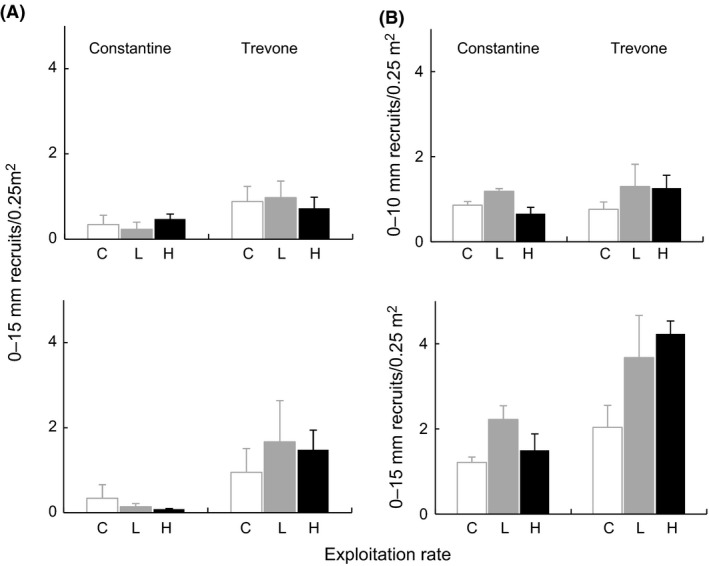
(A) Estimated mean numbers (±SE, *n *=* *3 plots per sample) of *Patella* spp. (0–15 mm) recruits scaled to sampled quadrat, at treatments plots (codes and locations as for Fig. 2 legend), in summer (top graph) and autumn (bottom graph) 1999. (B) Estimated mean numbers (±SE, *n *=* *3 plots per sample) of *Patella* spp. (0–10 mm) and (0–15 mm) recruits at the end of the experiment scaled to sampled quadrat, at treatment plots (codes and locations as for Fig. 2 legend).

No painted limpet was observed outside its original plot, indicating that the treatment effects impacted resident limpets within the plots. Recruits (0–15 mm) were considered to have arrived from the pelagic larval pool, subsequently emerging from nursery pools or crevices to use the space released by the removal of the larger limpets. No differences were detected (*P *>* *0.05) in estimated number of *Patella* spp. (0–15 mm) recruits in summer and autumn 1999, either under different levels of exploitation or between locations (Fig. 10A, ANOVA tables not presented).

At the end of the experiment, recruitment was assessed by grouping the removed 0–10 mm and 0–15 mm size classes (Fig. 10B). No differences were detected (*P *>* *0.05) at the end of the experiment in estimated number of *Patella* spp. 0–10 mm recruits either under different levels of exploitation or between locations. The abundance of 0–15 mm recruits differed by location, but no differences were detected between treatments (Fig. 10B, Table 5). Therefore, differences in recruitment by location at the end of the experiment were due to the 10–15 mm size class.

**Table 5:** Response of *Patella* spp. (0–15 mm) recruits at the end of the experiment by treatment and location (model 1: Factor codes as for Table 1). Terms with *P *<* *0.05 are in bold

Source	df	MS	*F*	*P*
Between subjects				
*L*′	1	16,261.10	8.01	**0.047**
*B*′(*L*′)	4	2028.90	–	
Within subjects				
* T*	2	4276.20	2.33	0.300
*T* × *L*′	2	1836.20	3.31	0.090
Residual error	8	555.40		

**Figure 11:**
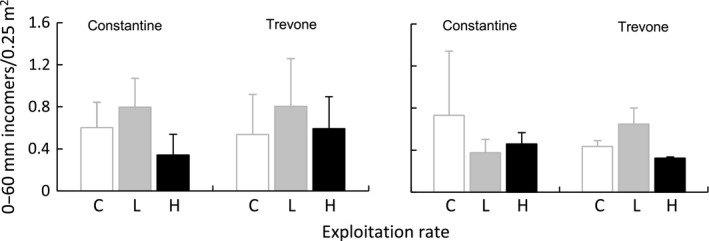
Estimated mean numbers (±SE, *n *=* *3 plots per sample) of *Patella* spp. (0–60 mm) incomers scaled to sampled quadrat, at treatments plots (codes and locations as for Fig. 2 legend), in summer (left graph) and autumn (right graph) 1999.

No differences were detected in the abundance of 0–60 mm new incomer individuals/0.25 m^2^ in summer and autumn 1999 (Fig. 11, Tables 6 and 7).

**Table 6:** Response of *Patella* spp. (0–60 mm) incomers at summer 1999 by treatment and location (model 1: Factor codes and orthogonal contrasts as for Table 1)

Source	df	Seq SS	Seq MS	*F*	*P*
Between subjects					
*L*′	1	0.02	0.02	0.10	0.768
*B*′(*L*′)	4	0.76	0.19	‐	
Within subjects					
*T*	2	0.35	0.18	0.93	0.426
NE vs. EX	1	0.02	0.02	0.09	0.770
Low vs. high	1	0.33	0.33	1.77	0.213
*L*′ × *T*	2	0.08	0.04	0.19	0.831
*L*′ × (NE vs. EX)	1	0.04	0.04	0.20	0.664
*L*′ × (low vs. high)	1	0.04	0.04	0.23	0.642
Residual error	8	1.72	0.21		

**Table 7:** Response of *Patella* spp. (0–60 mm) incomers at autumn 1999 by treatment and location (model 1: Factor codes and orthogonal contrasts as for Table 1)

Source	df	Seq SS	Seq MS	*F*	*P*
Between subjects					
*L*′	1	3.71	3.71	1.71	0.261
*B*′(*L*′)	4	8.67	2.17	–	
Within subjects					
*T*	2	0.16	0.08	0.15	0.866
NE vs. EX	1	0.16	0.16	0.15	0.765
Low vs. high	1	0.01	0.01	1.00	0.500
*L*′ × *T*	2	1.06	0.53	2.43	0.150
*L*′ × (NE vs. EX)	1	1.06	1.06	202.81	0.059
*L*′ × (low vs. high)	1	0.01	0.01	0.02	0.881
Residual error	8	1.75	0.22		

## Discussion

Fishing can lead to an evolutionary response in populations (Law 2000) or induce phenotypic plasticity (Reznick 1993) in some traits depending on its intensity, duration, and the biology of the species. Fishing on larger individuals of *Menidia menidia* exerted an evolutionary pressure for fish to be smaller and growth slower in just four generations (Conover and Munch 2002). For protogynic fishes with plastic sex change, size‐selective fishing practices can alter traits such as the mature population sex ratio and the timing of sexual transformation (Hamilton et al. 2007). Using historical data of *Semicossyphus pulcher* recreational and commercial fishing, Hamilton et al. (2007) found that where fishing intensified, males and females shifted significantly to smaller body sizes, females matured earlier and changed sex into males at both smaller sizes and younger ages and appeared to have a reduced maximum lifespan. In contrast, protogynic fish stocks with fixed sex change are predicted to be very sensitive to the size‐selective fishing pattern particularly if all male size classes are fished (Alonzo and Mangel 2004). In such cases, there is no possible sex‐ratio compensation for the removal of the males. However, for the former species, management efforts will benefit from the maintenance of sex ratio as well as stock size, with evaluations of recruitment based on sex ratio or male stock size in addition to the traditional female‐based stock–recruitment relationship (Heppell et al. 2006). Our results explore a plastic rather than an evolutionary response in patellid limpets drawn from the same pool of recruits from a panmictic larval population.

### Sexual maturity and size at sex change are context dependent

Reduction in mean sizes with increase of exploitation was observed in line with what occurs in most exploited stocks (e.g., Oliva and Castilla 1986; Pombo and Escofet 1996; Fenberg and Roy 2012) revealing that the treatments were effective (Figs. 4 and 6). Nevertheless, the mean sizes of different sexual stages depended on an interaction with exploitation, suggesting that different levels of exploitation will influence differentially the mean sizes of the sexual stages. Neuter shell mean size did not vary much under different exploitation rates, but males and females mean size both decreased with exploitation (Fig. 3). This suggests that there were no direct (due to removal) or indirect (via prematurely switch of neuters to males) effects of exploitation (hence reduction in density of larger size classes) on the shell mean size of neuters.

Mean sizes of neuters were, however, significantly smaller than mean sizes of males and females, and mean sizes of males were also significantly smaller than those of females. This is in accordance with what happens in natural populations (Orton et al. 1956; Ballantine 1961; Baxter 1983). Irrespective of the exploitation regime and probably due to the occurrence of sex change, limpet populations exhibited the expected distribution of sexual stages throughout the size ranges: Neuters were smaller than males, and males were smaller than females.

There was strong evidence that size at sex change decreased in response to exploitation, given by analysis of the averaged *L*_50_ extracted from the logistic regression on each treatment plot (Fig. 4). As shown by the GLM, the removal of limpets had increased the probability of being female with intensification of treatment level. This suggests that with the removal of the mainly female larger size classes, males were compensating that loss through earlier and more frequent sex change. This sex change was happening, as in natural populations, at larger sizes of males as an increase in size also increased the probability of being female. The investigation of the relative size of sex change (*L*_50_ */L*_max_) indicated that regardless of exploitation these limpet populations were changing sex at 90% of their maximum size (Fig. 7). Le Quesne and Hawkins (2006) indicated 15–25 mm as the likely size‐range over which sex change might occur for the *P. vulgata* population from their study based on the size class overlap. Sundelöf et al. (2010) when investigating determinants of reproductive potential in *P. vulgata* considered that the transition from male to females occurred at an age comprised between 3 and 6 years as previously described by Orton et al. (1956) and Ballantine (1961). Determining the age for the 50.5 ± 6.4 mm (averaged *L*_50_ of control plots) sex‐changing limpets would have contributed usefully to the knowledge of sex change in this study, but the limpet shells do not exhibit annual growth rings.

No differences were detected in the densities of different sexual stages, suggesting some mechanism that compensated for the loss of the larger individuals, which could be recruitment combined with sex change. Males changed earlier in size to females, and it is possible that larger neuters were also compensating for sex‐changing males, by changing to males prematurely and themselves being compensated by recruitment. The significant stage × location interaction and a corresponding significant (NS vs. SE) contrast × location interaction indicated differences in numbers of the not sexed (neuters) and sexed individuals by location (Fig. 5, Table 3). Neuters were essentially immature small individuals more abundant at Trevone than at Constantine: At the end of the experiment, 0–15 mm recruits were more abundant at Trevone (Fig. 10B, Table 5). With the removal of the bigger limpets, recruits had more space to move and forage, but since Trevone is a flatter shore than Constantine with fewer crevices allowing refuge for smaller immature individuals, recruitment was more apparent at Trevone.

No significant differences were detected in *P. vulgata* growth (Fig. 8), or in the microalgal food available (Fig. 9) despite the reduction of grazing pressure on microalgal growth. The absence of differential growth with exploitation could be the result of energy being allocated to the observed early sexual maturity and anticipation in size of sex change of males to female rather than growth. There were, however, indications of greater microalgal growth at Trevone. Reductions in limpet numbers, with consequent reduced grazing pressure, could influence the promotion of algal growth by limpet mucus (see Davies et al. 1992). The interaction of low‐versus‐high exploitation contrasts with location and distance to the microalgal food available at the plot center and edge, and also of exploitation level with location at the end of the experiment (Table 4), suggested that other factors could be determining micro‐food abundance (see Thompson et al. 2005). These could include grazing activity due to recruitment or migration of other grazers, and exposure to desiccation of treatment plots (due to height on the shore and shore hydrodynamics). Nevertheless, food availability was probably not a limiting factor inhibiting progression from male to female, as the release of bare rock with the removal of the larger limpets is likely to have made more food available for the smaller size classes (Boaventura et al. 2003).

However, the experiment did not segregate the effects of density and sex ratio. Both have been shown to influence sex change in the Patellogastropoda (see Wright and Lindberg 1982; Lindberg and Wright 1985; Wright 1989; Collin 2013), and as most individuals were not followed from the beginning of the experiment, these results must be interpreted with caution. Nevertheless, we hope they will provide insights for future manipulative experiments in sex‐changing limpets.

Our research indicates that sex change in *P. vulgata* can occur at smaller sizes following reductions in density of the largest size classes. These results are consistent with those shown by the protandric *P. ferruginea* where individuals in populations with low density of larger individuals switch to female at smaller sizes (Rivera‐Ingraham et al. 2011).

### Possible pathways of sexual identity in individual limpets

**Figure 12:**
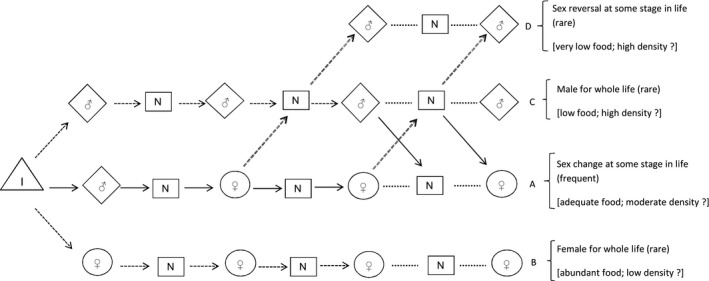
Hypothetical pathways of sexual identity in protandric individual limpets with a resting phase, such as *Patella vulgata* (I, immature; N, neuters; ♂, males; ♀, females). Arrows with dotted lines indicate less frequent pathways; dotted lines without arrow heads indicate subsequent stages as same sex alternating with resting stages for remainder of life; possible environmental conditions promoting each pathway are presented in square brackets.

In limpet populations with a neuter resting phase between breeding seasons such as *P. vulgata,* there are several possibilities of pathways of sex throughout life of an individual. These alternative sequences are summarized in Fig. 12. The proportions and sizes of sexes in populations of protandric limpets suggest that most individuals will change sex from male to female via a neuter resting phase (for histological and endocrinological data see Choquet 1971) (protandric sex‐change pathway A). Some might be less frequent such as direct maturation from immature to female, or sex reversal rather than unidirectional protandric sex change (Le Quesne and Hawkins 2006; Guallart et al. 2013). At lower densities, possibly with more food available, this occurs earlier (see also Lindberg and Wright 1985; Wright 1989) and could help to correct skewed sex ratios (Fenberg and Roy 2012; Rivera‐Ingraham et al. 2011). Occasional small females are also found (Borges 2013), suggesting that some individuals proceed directly to female at first maturity as occurs in nonprotandric species; this is probably rare but occurs in populations with low densities with abundant food to provide the necessary energy for female differentiation (no sex‐change pathway B, female for whole life). Some individuals remain male throughout their lives as indicated by occasional large males (see Baxter 1983; Borges 2013; Guallart et al. 2013) (C: no sex‐change pathway, male for whole life). Large males could also be the outcome of sex reversal to male by previously female individuals after a resting phase (D: sex reversal pathway) as shown by Le Quesne and Hawkins (2006) in *P. vulgata* and Guallart et al. (2013) in *P. ferruginea*. The incidence of remaining male or sex reversal is probably rare, occurring under high densities, where very low food would constrain viability of females, usually requiring more energy than males (see Warner 1988), and/or as a mechanism to compensate the reduction in numbers of males. In populations subjected to size‐selective exploitation, where most of the bigger females are removed, these frequencies of sex direction will most likely change in order to compensate for the losses of the females and the sex change will occur at smaller sizes than under no exploitation (Rivera‐Ingraham, et al. 2011; Fenberg and Roy 2012). However, in order to test these predictions, tagged individuals of known sex from biopsy must be followed in both natural and simulated conditions.

### Implications for conservation

Reproductive output of *P. vulgata* seems more sensitive to perturbations in the survival of large males and medium and large females than to perturbations in recruitment (Sundelöf et al. 2010). Hence, the removal of the larger sex‐changing limpets by collectors seriously compromises population persistence, particularly over short timescales if recruitment is low. In protandric species with sex‐change plasticity, the depletion of larger individuals could, however, be partially compensated if individuals are allowed to differentiate as male and later to switch to female (Rivera‐Ingraham et al. 2011; our study). Knowledge of sex‐change dynamics in such populations would allow management procedures to counteract population decline and is essential to resource management and conservation on rocky shores. A precautionary approach would involve having both a minimum size (to ensure breeding individuals are present in the population) and a maximum size (to ensure some large females remain).

## Conflict of Interest

None declared.

## References

[ece31872-bib-0001] Aguilera, M. A. , and S. A. Navarrete . 2007 Effects of *Chiton granosus* (Frembly, 1827) and other molluscan grazers on algal succession in wave exposed mid‐intertidal rocky shores of central Chile. J. Exp. Mar. Biol. Ecol. 349:84–98.

[ece31872-bib-0002] Allsop, D. J. 2003 The evolutionary ecology of sex change. [Ph.D. thesis], University of Edinburgh, Edinburgh, UK.

[ece31872-bib-0003] Allsop, D. J. , and S. A. West . 2003 Constant relative age and size at sex change for sequentially hermaphroditic fish. J. Evol. Biol. 16:921–929. 14635907 10.1046/j.1420-9101.2003.00590.x

[ece31872-bib-0004] Allsop, D. J. , and S. A. West . 2004 Sex‐ratio evolution in sex changing animals. Evolution 58:1019–1027. 15212382 10.1111/j.0014-3820.2004.tb00435.x

[ece31872-bib-0005] Alonzo, S. H. , and M. Mangel . 2004 The effects of size‐selective fisheries on the stock dynamics of and sperm limitation in sex‐changing fish. Fish. Bull. 102:1–13.

[ece31872-bib-0006] Alonzo, S. H. , and M. Mangel . 2005 Sex‐change rules, stock dynamics, and the performance of spawning‐per‐recruit measures in protogynous stocks. Fish. Bull. 103:229–245.

[ece31872-bib-0007] Álvarez, M. , I. B. Godino , A. Balbo , and M. Madella . 2011 Shell middens as archives of past environments, human dispersal and specialized resource management. Quat. Int. 239:1–7.

[ece31872-bib-0008] Bacci, G. 1975 Sex reversal in the genus *Patella* (Gastropoda, Prosobranchiata). Publicazioni della Stazione Zoologica di Napoli 39:341–344.

[ece31872-bib-0009] Baldwin, A. P. , and R. T. Bauer . 2003 Growth, survivorship, life‐span, and sex change in the hermaphroditic shrimp *Lysmata wurdemanni* (Decapoda: Caridea: Hippolytidae). Mar. Biol. 143:157–166.

[ece31872-bib-0010] Ballantine, W. J. (1961) The population dynamics of Patella vulgata and other limpets. [Ph.D. thesis], Queen Mary College, London University, London.

[ece31872-bib-0011] Baskett, M. L. , S. A. Levin , S. D. Gaines , and J. Dushoff . 2005 Marine reserve design and the evolution of size at maturation in harvested fish. Ecol. Appl. 15:882–901.

[ece31872-bib-0012] Baxter, J. M. 1983 Annual variations in soft‐body dry weight, reproductive‐cycle and sex‐ratios in populations of *Patella vulgata* at adjacent sites in the Orkney islands. Mar. Biol. 76:149–157.

[ece31872-bib-0013] Boaventura, D. , L. Cancela da Fonseca , and S. J. Hawkins . 2003 Size matters: competition within populations of the limpet *Patella depressa* . J. Anim. Ecol. 72:435–446.

[ece31872-bib-0014] Borges, C. D. G. 2013 The influence of environment and exploitation on sex change in limpets. [Ph.D. thesis], University of Southampton, Southampton, UK.

[ece31872-bib-0015] Buxton, C. D. 1993 Life‐history changes in exploited reef fishes on the east coast of South Africa. Environ. Biol. Fishes 36:47–63.

[ece31872-bib-0016] Catterall, C. P. , and I. R. Pointer . 1987 The potential impact of human gathering on shellfish populations, with reference to some NE Australia intertidal flats. Oikos 50:14–122.

[ece31872-bib-0100] Castilla, J. C. , and L. R. Duran . 1985 Human Exclusion from the Rocky Intertidal Zone of Central Chile: The Effects on Concholepas Concholepas (Gastropoda). Oikos 45:391–399.

[ece31872-bib-0017] Choquet, M. 1971 Etude do cycle biologique et de l'inversion du sexe chez *Patella vulgata* L. (Mollusque Gastéropode Prosobranche). Gen. Comp. Endocrinol. 16:59–73. 5542997 10.1016/0016-6480(71)90208-5

[ece31872-bib-0018] Coleman, R. A. , A. J. Underwood , L. Benedetti‐Cecchi , P. Ålberg , F. Arenas , J. Arrontes , et al. 2006 A continental scale evaluation of the role of limpet grazing on rocky shores. Oecologia 147:556–564. 16450182 10.1007/s00442-005-0296-9

[ece31872-bib-0019] Collin, R. 2006 Sex ratio, life history invariants, and patterns of sex change in a family of protandrous gastropods. Evolution 60:735–745. 16739455

[ece31872-bib-0020] Collin, R. 2013 Phylogenetic patterns and phenotypic plasticity of molluscan sexual systems. Integr. Comp. Biol. 53:723–735. 23784696 10.1093/icb/ict076

[ece31872-bib-0021] Conover, D. O. , and S. B. Munch . 2002 Sustaining fisheries yields over evolutionary time scales. Sciences 297:94–96. 10.1126/science.1074085 12098697

[ece31872-bib-0022] Corte‐Real, H. B. S. M. , S. J. Hawkins , and J. P. Thorpe . 1996 Population differentiation and taxonomic status of the exploited limpet *Patella candei* in the Macaronesian islands (Azores, Madeira, Canaries). Mar. Biol. 125:141–152.

[ece31872-bib-0023] Crowe, T. P. , N. J. Frost , and S. J. Hawkins . 2011 Interactive effects of losing key grazers and ecosystem engineers vary with environmental context. Mar. Ecol. Prog. Ser. 430:223–234.

[ece31872-bib-0024] Davies, M. S. , S. J. Hawkins , and H. D. Jones . 1992 Pedal mucus and its influence on the microbial food‐supply of two intertidal gastropods, *Patella vulgata* L. and *Littorina littorea* (L). J. Exp. Mar. Biol. Ecol. 161:57–77.

[ece31872-bib-0025] Davies, A. J. , M. P. Johnson , and C. A. Maggs . 2007 Limpet grazing and loss of *Ascophyllum nodosum* on decadal time scales. Mar. Ecol. Prog. Ser. 339:131–141.

[ece31872-bib-0026] DeMartini, E. E. , A. M. Friedlander , and S. R. Holzwarth . 2005 Size at sex change in protogynous labroids, prey body size distributions, and apex predator densities at NW Hawaiian atolls. Mar. Ecol. Prog. Ser. 297:259–271.

[ece31872-bib-0027] Dodd, J. M. 1956 Studies on the biology of limpets. III. Hermaphroditism in the three British species of *Patella* . J. Mar. Biol. Assoc. U.K. 35:327–340.

[ece31872-bib-0028] Doncaster, C. P. , and A. J. H. Davey . 2007 Analysis of variance and covariance. How to choose and construct models for the life sciences. Cambridge Univ. Press, Cambridge.

[ece31872-bib-0029] Duran, L. R. , and J. C. Castilla . 1989 Variation and persistence of the middle rocky intertidal community of central Chile, with and without human harvesting. Mar. Biol. 103:555–562.

[ece31872-bib-0030] Eekhout, S. , C. M. Raubenheimer , G. M. Branch , A. L. Bosman , and M. O. Bergh . 1992 A holistic approach to the exploitation of intertidal stocks – limpets as a case study. S. Afr. J. Mar. Sci. 12:1017–1029.

[ece31872-bib-0031] Espinosa, F. , G. Rivera‐Ingraham , and J. C. García‐Gómez . 2009a Gonochorism or protandrous hermaphroditism? Evidence of sex change in the endangered limpet *Patella ferruginea* . Mar Biodivers Rec 2:e153.

[ece31872-bib-0032] Espinosa, F. , G. A. Rivera‐Ingraham , D. Fa , and J. C. García‐Gómez . 2009b Effect of human pressure on population size structures of the endangered ferruginean limpet: toward future management measures. J. Coastal Res. 25:857–863.

[ece31872-bib-0033] Fenberg, P. B. , and K. Roy . 2008 Ecological and evolutionary consequences of size‐selective harvesting: how much do we know*?* Mol. Ecol. 17:209–220. 17868288 10.1111/j.1365-294X.2007.03522.x

[ece31872-bib-0034] Fenberg, P. B. , and K. Roy . 2012 Anthropogenic harvesting pressure and changes in life history: insights from a rocky intertidal limpet. Am. Nat. 180:200–210. 22766931 10.1086/666613

[ece31872-bib-0035] Fennessy, S. , and Y. Sadovy . 2002 Reproductive biology of a diandric protogynous hermaphrodite, the serranid *Epinephelus andersoni* . Mar. Freshw. Res. 53:147–158.

[ece31872-bib-0036] Ghiselin, M. T. 1969 The evolution of hermaphroditism among animals. Q. Rev. Biol. 44:189–208. 4901396 10.1086/406066

[ece31872-bib-0037] de Girolamo, M. , M. Scaggiante , and M. B. Rasotto . 1999 Social organization and sexual pattern in the Mediterranean parrotfish *Sparisoma cretense* (Teleostei: Scaridae). Mar. Biol. 135:353–360.

[ece31872-bib-0038] Guallart, J. , M. Calvo , I. Acevedo , and J. Templado . 2013 Two‐way sex change in the endangered limpet *Patella ferruginea* (Mollusca, Gastropoda). Invertebr. Reprod. Dev. 57:247–253.

[ece31872-bib-0039] Hamilton, S. L. , J. E. Caselle , J. D. Standish , D. M. Schroeder , M. S. Love , J. A. Rosales‐Casian , et al. 2007 Size‐selective harvesting alters life histories of a sex‐changing fish. Ecol. Appl. 17:2268–2280. 18213967 10.1890/06-1930.1

[ece31872-bib-0040] Hannah, R. W. , and S. A. Jones . 1991 Fishery‐induced changes in the population structure of pink shrimp (*Pandalus jordani*). Fish. Bull. 89:41–51.

[ece31872-bib-0041] Hawkins, S. J. , and R. G. Hartnoll . 1983 Grazing of intertidal algae by marine invertebrates. Oceanogr. Mar. Biol. 21:195–282.

[ece31872-bib-0042] Hawkins, J. P. , and C. M. Roberts . 2003 Effects of fishing on sex‐changing Caribbean parrotfishes. Biol. Conserv. 115:213–226.

[ece31872-bib-0043] Hawkins, S. J. , D. C. Watson , A. S. Hill , S. P. Harding , M. A. Kyriakides , S. Hutchinson , et al. 1989 A comparison of feeding mechanisms in microphagous herbivorous intertidal prosobranchs in relation to resource partitioning. J. Molluscan Stud. 55:151–165.

[ece31872-bib-0044] Hawkins, S. J. , R. G. Hartnoll , J. M. Kain , and T. A. Norton . 1992 Plant‐animal interactions on hard substratum the North‐West Atlantic Pp. l–32 *in* John D. M., Hawkins S. J., and Price J. H., eds. Plant‐animal interactions in the marine benthos. Clarendon Press, Oxford.

[ece31872-bib-0045] Hawkins, S. J. , H. B. S. M. Corte‐Real , F. G. Pannacciulli , L. C. Weber , and J. D. D. Bishop . 2000 Thoughts on the ecology and evolution of the intertidal biota of the Azores and other Atlantic islands. Hydrobiologia 440:3–17.

[ece31872-bib-0046] Heppell, S. S. , S. A. Heppell , F. C. Coleman , and C. C. Koenig . 2006 Models to compare management options for a protogynous fish. Ecol. Appl. 16:238–249. 16705976 10.1890/04-1113

[ece31872-bib-0047] Hockey, P. A. R. , A. L. Bosman , and W. R. Siegfried . 1988 Patterns and correlates of shellfish exploitation by coastal people in Transkei: an enigma of protein production. J. Appl. Ecol. 25:353–363.

[ece31872-bib-0048] Jenkins, S. R. , R. A. Coleman , P. Della Santina , S. J. Hawkins , M. T. Burrows , and R. G. Hartnoll . 2005 Regional scale differences in the determinism of grazing effects in the rocky intertidal. Mar. Ecol. Prog. Ser. 287:77–86.

[ece31872-bib-0049] Lasiak, T. 1991 The susceptibility and/or resilience of rocky littoral molluscs to stock depletion by the indigenous coastal people of Transkei, southern Africa. Biol. Conserv. 56:245–264.

[ece31872-bib-0050] Lasiak, T. 1992 Contemporary shellfish‐ gathering practices of indigenous coastal people in Transkei: some implications for interpretation in the archaeological record. S. Afr. J. Sci. 88:19–28.

[ece31872-bib-0051] Law, R. 2000 Fishing, selection, and phenotypic evolution. ICES J. Mar. Sci. 57:659–668.

[ece31872-bib-0052] Le Quesne, W. J. F. , and S. J. Hawkins . 2006 Direct observations of protandrous sex change in the patellid limpet *Patella vulgata* . J. Mar. Biol. Assoc. U.K. 86:161–162.

[ece31872-bib-0053] Lindberg, D. R. , and W. G. Wright . 1985 Patterns of sex change of the protandric patellacean limpet *Lottia gigantea* (Mollusca: Gastropoda). Veliger 27:261–265.

[ece31872-bib-0054] McLachlan, A. , and H. W. Lombard . 1981 Growth and production in exploited and unexploited populations of a rocky shore gastropod, *Turbo sarmaticus* . Veliger 23:221–229.

[ece31872-bib-0055] Montalenti, G. 1958 Perspectives of research on sex problems in marine organisms Pp. 589–602 *in* Buzzati‐Travesso A. A., ed. Perspectives in marine biology. Univ. of California Press, Berkeley.

[ece31872-bib-0056] Moreno, C. A. 2001 Community patterns generated by human harvesting on Chilean shores: a review. Aquat. Conserv. 11:19–30.

[ece31872-bib-0057] Moreno, C. , K. M. Lucnecke , and M. I. Lepez . 1986 The response of an intertidal *C. concholepas* (Gasteropoda) population to protection from Man in Southern Chile and the effects on benthic sessile assemblages. Oikos 42:354–364.

[ece31872-bib-0058] Munday, P. L. , P. M. Buston , and R. R. Warner . 2006 Diversity and flexibility of sex‐change strategies in animals. Trends Ecol. Evol. 21:89–95. 16701480 10.1016/j.tree.2005.10.020

[ece31872-bib-0059] Oliva, D. , and J. C. Castilla . 1986 The effect of human exclusion on the population‐structure of key‐hole limpets *Fissurella crassa* and *Fissurella limbata* on the coast of central Chile. Mar. Ecol. 7:201–217.

[ece31872-bib-0060] Ortega, S. 1987 The effect of human gathering on the size distribution of *Siphonaria gigas* (Mollusca, Pulmonata) on the Pacific coast of Costa Rica. Veliger 29:51–255.

[ece31872-bib-0061] Orton, J. H. 1919 Sex phenomena in the common limpet (*Patella vulgata*). Nature 104:373–374.

[ece31872-bib-0062] Orton, J. H. , A. J. Southward , and J. M. Dodd . 1956 Studies on the biology of limpets. II. The breeding of *Patella vulgata* L. in Britain. J. Mar. Biol. Assoc. U.K. 35:149–176.

[ece31872-bib-0063] Platten, J. R. , I. R. Tibbetts , and M. J. Sheaves . 2002 The influence of increased line‐fishing mortality in the sex ratio and age of sex reversal of the venus tusk fish. J. Fish Biol. 60:301–318.

[ece31872-bib-0064] Pombo, O. A. , and A. Escofet . 1996 Effect of exploitation on the limpet *Lottia gigantea:* a field study in Baja California (Mexico) and California (U.S.A.). Pac. Sci. 50:393–403.

[ece31872-bib-0200] R Core Team . 2015 R: A Language and Environment for Statistical Computing. R Foundation for Statistical Computing, Vienna, Austria, https://www.R-project.org.

[ece31872-bib-0065] Reznick, D. N. 1993 Norms of reaction in fishes Pp. 72–90 *in* Stokes T. K., McGlade J. M., and Law R., eds. The exploitation of evolving resources. Springer‐Verlag, Berlin.

[ece31872-bib-0066] Rivera‐Ingraham, G. A. , F. Espinosa , and J. C. García‐Gómez . 2011 Environmentally mediated sex change in the endangered limpet *Patella ferruginea* (Gastropoda: Patellidae). J. Molluscan Stud. 77:226–231.

[ece31872-bib-0067] Santos, R. S. , S. J. Hawkins , L. R. Monteiro , M. Alves , and E. J. Isidro . 1995 Marine research, resources and conservation in the Azores. Aquat. Conserv. 5:311–354.

[ece31872-bib-0068] Sato, T. , and S. Goshima . 2006 Impacts of male‐only fishing and sperm limitation in manipulations of an unfished crab, *Hapalogaster dentata* . Mar. Ecol. Prog. Ser. 313:193–204.

[ece31872-bib-0069] Sokal, R. R. , and F. J. Rohlf (1998) Biometry: the principles and practice of statistics in biological research. W.H. Freeman and Company, New York.

[ece31872-bib-0070] Steele, T. E. , and R. G. Klein . 2008 Intertidal shellfish use during the Middle and Later Stone Age of South Africa. Archaeofauna 17:63–76.

[ece31872-bib-0071] Sundelöf, A. , S. R. Jenkins , C. J. Svensson , J. Delany , S. J. Hawkins , and P. Ålberg . 2010 Determinants of reproductive potential and population size in open populations of *Patella vulgata* . Mar. Biol. 157:779–789.

[ece31872-bib-0072] Thompson, G. B. 1979 Distribution and population dynamics of the limpet *Patella ulyssiponensis* in Bantry Bay. J. Exp. Mar. Biol. Ecol. 40:115–135.

[ece31872-bib-0073] Thompson, R. C. , M. L. Tobin , S. J. Hawkins , and T. A. Norton . 1999 Problems in extraction and spectrophotometric determination of chlorophyll from epilithic microbial biofilms: towards a standard method. J. Mar. Biol. Assoc. U.K. 79:551–558.

[ece31872-bib-0074] Thompson, R. C. , P. S. Moschella , S. R. Jenkins , T. A. Norton , and S. J. Hawkins . 2005 Differences in photosynthetic marine biofilms between sheltered and moderately exposed rocky shores. Mar. Ecol. Prog. Ser. 296:53–63.

[ece31872-bib-0075] Underwood, A. J. 1997 Experiments in ecology. Cambridge Univ. Press, Cambridge.

[ece31872-bib-0076] Warner, R. R. 1988 Sex change and the size‐advantage model. Trends Ecol. Evol. 3:133–136. 21227182 10.1016/0169-5347(88)90176-0

[ece31872-bib-0077] Warner, R. R. , D. L. Fitch , and J. D. Standish . 1996 Social control of sex change in the shelf limpet, *Crepidula norrisiarum*: size‐specific responses to local group composition. J. Exp. Mar. Biol. Ecol. 204:155–167.

[ece31872-bib-0078] Wright, W. G. 1989 Intraspecific density mediates sex‐change in territorial patellacean *Lottia gigantea* . Mar. Biol. 100:353–364.

[ece31872-bib-0079] Wright, W. G. , and D. R. Lindberg . 1982 Direct observations of sex change in the patelacean *Lottia gigantea* . J. Mar. Biol. Assoc. U.K. 62:737–738.

